# The *Opuntia effect* and the Reactivation of Ovarian Function and Blood Metabolite Concentrations of Anestrous Goats Exposed to Active Males

**DOI:** 10.3390/ani9080550

**Published:** 2019-08-13

**Authors:** Cesar A. Meza-Herrera, Carlos A. Romero-Rodríguez, Adrian Nevárez-Dominguez, Arnoldo Flores-Hernández, Omag Cano-Villegas, Ulises Macías-Cruz, Miguel Mellado, Guadalupe Calderón-Leyva, Dalia Carrillo-Moreno, Francisco G. Véliz-Deras

**Affiliations:** 1 Unidad Regional Universitaria de Zonas Áridas, Programa de Posgrado, Universidad Autónoma Chapingo, Bermejillo, Durango 35230, Mexico; 2 Instituto de Estudios de Posgrado, Universidad de Córdoba, 14014 Córdoba, Spain; 3 Facultad de Ciencias Biológicas, Universidad Juárez del Estado de Durango, Gómez Palacio, Durango 35010, Mexico; 4 Instituto de Ciencias Agrícolas, Universidad Autónoma de Baja California, Mexicali 21705, Mexico; 5 Departamento de Ciencias Médico Veterinarias, Universidad Autónoma Agraria Antonio Narro, Unidad Laguna, Periférico Raúl López Sánchez y Carretera a Santa Fe, Torreón, Coahuila 27054, Mexico

**Keywords:** goats, male effect, *Opuntia*, targeted supplementation, reproductive efficiency

## Abstract

**Simple Summary:**

We evaluated the potential supplementation effect of protein enriched *Opuntia* cladodes, the flat leaf-like steam of cactus species (PEO), upon changes of blood metabolites, estrus induction, estrus latency, and ovulation rate in anestrous goats exposed to sexually active males. We observed that PEO positively influenced reproductive outcomes without changes in blood metabolites. Our results highlight the importance that bio-fortified *Opuntia* cladodes supplementation exerts on anestrous goats exposed to the male effect as an option to improve not only the out-of-season reproductive efficiency of goats but to enhance the sustainability of marginal, extensive and semi-arid goat production systems.

**Abstract:**

The effect of protein enriched *Opuntia* cladodes supplementation upon changes of serum total protein, urea, cholesterol, glucose as related to estrus induction (EI%), estrus latency (EL, h), and ovulation rate (OR, units) in adult anestrous goats exposed to the male effect was evaluated. In late April, anestrus goats (*n* = 45, 25° N) homogeneous regarding live weight (LE; 43.8 ± 1.6 kg) and body condition score (BCS; 2.3 ± 0.1 units) were randomly assigned to: (1). ***Protein-enriched Opuntia*** (**PEO**; *n* = 15; 29.8% CP, 2.2 Mcal ME kg^−1^), (2). ***Non-enriched Opuntia*** (**NEO**; *n* = 15; 6.4% CP, 2.1 Mcal ME kg^−1^), and (3). ***Control*** (**CON**; *n* = 15). NEO and PEO goats were individually supplemented with cladodes (160 g d^−1^; 0900–1000 h), thereafter all groups grazed in a marginal rangeland (1000–1800 h). Neither LW (*p* > 0.05) nor BCS (*p* > 0.05) differed among groups, yet an increased (*p* < 0.05) EI % (100, 57, 42 ± 0.16%), EL h (62, 60, 32 ± 4.2 h), and OR (1.33, 0.71, 0.43 ± 0.23 units) occurred in PEO and NEO vs. CONT, respectively. However, neither blood metabolites differed among groups nor a treatment x time interaction occurred. Peri-breeding protein enriched *Opuntia* cladodes supplementation of anestrous goats exposed to active males increased (*p* < 0.01) reproductive outcomes during the non-breeding season.

## 1. Introduction

Worldwide, arid and semi-arid lands besides those characterized by scarce water availability, drought, overgrazing, and poor quality soils, while being linked to vulnerable human livelihoods, they also concentrate an important percentage of the goat inventory [1,2]. Such a scenario of limited biotic and economic resources in the dry-lands generates conditions prone to the establishment of highly susceptible goat production systems that are greatly dependent on communal rangelands with limited productivity [1,3]. Despite this complex scenario, goats not only survive but even flourish under such compromised environments; they show remarkable physiological plasticity, adaptation capacity, reproductive performance, and productive longevity, contributing in a significant fashion to the sustainability of goat producers under marginal production schemes [1,4]. 

In these low-input goat production systems, the use of commercial nutritional concentrates or high quality forages is almost negligible, a situation that can compromise the sustainability of these production systems. Therefore, cost-effective and productive-viable supplementation alternatives must be developed [1,5]. In this context, the use of diverse agro-industrial by-products, nutritional blocks, forage bushes, or shrubs, and even low-cost alternative supplements (i.e., by-pass protein, fat by-pass, glutamate or betacarotene) could be important options to speed up goat productive–reproductive performance [6,7,8,9,10]. Furthermore, most goat production systems under arid and semi-arid conditions are positively associated to the presence of native cacti (*Opuntia* spp) that has a high potential as alternative forage besides its high content of polyphenols, vitamins, polyunsaturated fatty acids, and amino acids [11]. Nonetheless, even though the *Opuntia* cladodes, the flat leaf-like stem of cactus species, have an increased level of calcium and carbohydrates, the content of both fiber and crude protein (CP) is low [12,13]. In this respect, while increases up to 12.8% in CP have been reported throughout a protein-enrichment of *Opuntia* cladodes process with *Aspergillus niger* [14], other CP upturns from 4% to 30% [15], while significant increases in CP (i.e., 400%; 260 g/kg dry matter) was reported when cladodes were exposed to a fermentation process with *Saccharomyces cerevisiae* [16]. 

Due to the biological and marketing constrains generated by the seasonal reproduction pattern depicted by goats under temperate and subtropical conditions, different estrus-induction hormonal based protocols to reduce the exogenous-hormone load have been recently developed [17]. In addition, the use of not only socio-sexual cues (i.e., the male and female effects), but also targeted nutritional supplementation to increase certain blood metabolites and/or metabolic hormones positively linked to reproductive outputs, are both interesting strategies to activate the hypothalamous-pituitary-gonadal axis (HPG) to induce out-of-season ovarian cyclicity in small ruminants [18,19,20]. Furthermore, the female response to the male effect has been tightly modulated by the nutritional status, observing a positive relationship between nutritional supplementation and the efficacy of the male effect, affecting in a paramount fashion the reproductive performance [18,19]. Building on such findings, we tested the hypothesis that focus supplementation of protein enriched *Opuntia megacantha Salm-Dyck* cladodes would induce the ovarian function with increases in selected blood metabolites, enhancing the reproductive outcomes in previously anestrous adult goats exposed to the male effect and managed under marginal rangeland-grazing conditions. 

## 2. Material and Methods

### 2.1. General

All procedures and methods used in this study regarding the use and care of animals were carried-out in strict accordance with accepted international [21] and national [22] animal use and care guidelines, with institutional approval UACH-DGIP-REBIZA-16-510-4006.

### 2.2. Location, Environmental and Rangeland Conditions, Animal Care

The study was carried out in a commercial farm managed under extensive conditions sited in the “6 de enero” locality, Lerdo, Durango, Northern Mexico (25°31′ N, 103°36′ W, 1148 m elevation). While the rainy season extends from June to October, the mean annual rainfall and temperature are 225 mm and 24 °C, respectively. Additionally, whereas the relative humidity varies from 26.1% to 60.7%, the photoperiod ranges from 13 h 41 min (summer solstice, June) to 10 h 19 min (winter solstice, December). Interestingly, this Goat Production Unit is located close to the margins of the Nazas River, therefore, this lower basin is an interesting agroecosystem with a very special microenvironment from a soil quality and humidity level stand point. Certainly, this area has large spaces devoted to forage production (i.e., alfalfa, sorghum forage, and corn forage) with a huge availability of agricultural by-products and crop residues. 

Besides that, the other component of this complex agroecosystem includes the rangeland constituent, which has a vegetation characterized as Chihuahuan desert rangeland, previously described [23]. Briefly, while creosotebush (*Larrea tridentata* (*DC. Cov*) leads the grazing area, other key species include lechuguilla (*Agave lechuguilla Torr*), mesquite (*Prosopis glandulosa v. glandulosa*), and blue gramma (*Bouteloua gracilis* (*Wild*). *Ex Kunth Lag. Ex Griffiths*) [23]. Goats mostly graze on rangelands, although they have a stress-free access to crop residues such as corn, sorghum, cotton by-products, and alfalfa because of being located in the margins of the Nazas River, this Goat Production Unit is located inside the Irrigation District established in this area. Goats walk approximately 5 km daily from the pen to diverse sites of the available rangeland, therefore grazing restrains can be considered trifling [23]. During the spring-summer seasons, goats grazed the rangeland driven by a herdsman nine hours daily (1000 to 1900 h) and penned from 1900 to 1000 h. Goats spent the night in unroofed corral where they had free access to water and a commercial mineral-mix. 

### 2.3. Animals and Experimental Treatments

Mix-breed adult non-pregnant, non-lactating, anestrous goats (Criollo × Alpine-Saanen-Nubian; *n* = 45, multiparous, 3–4 yr. old) of known fertility were kept isolated from sight, sound, and smell of bucks at least three months before the onset of the trial (15 February). Thereafter, during the mid-anestrous season (20 April), goats were randomly distributed into three experimental groups: (1). ***Protein enriched Opuntia*** (**PEO**; *n* = 15; 44.6 ± 1.5 kg LW, 2.58 ± 0.13 units BCS), (2). ***Non-enriched Opuntia*** (**NEO**; *n* = 15; 43.9 ± 1.7 kg LW, 2.57 ± 0.12 units BCS) and (3). ***Control*** (**CC**; *n* = 15; 45.1 ± 1.6 kg LW, 2.57 ± 0.13 units, BCS), without feed supplementation. Both the NEO and PEO goats were individually supplemented with 160 g day^−1^ from 0900 to 1000 h during a 10 d adaptation period (20–30 April). Such supplementation schedule was based on previous observations where goats consumed the entire supplement if offered prior to grazing [24]. Goats had free access to water and a commercial mineral-mix at the pen during the evening-night hours, and were not treated against internal parasites, since this is not a common health problem under this dry environment.

### 2.4. Experimental Supplements and Supplementation Schedule 

The experimental group PEO considered the protein-enrichment of cladodes throughout a semisolid fermentative process by mixing small slices of *Opuntia* cladodes inoculated with *Scharomyces cereveciae* (1%), urea (1%) and ammonium sulphate (0.1%) in a bioreactor (NOPAFER-R, No. 2641-IMPI, Mexico) during a period of 10 h. Thereafter, the enriched cladodes were semi-dried at ambient temperature during 72 h. *Opuntia* supplementation in both the NEO and PEO experimental groups was from 0900 to 1000 h. The chemical composition of both *Opuntia* treatments (NEO and PEO) is presented in Table 1. The three experimental groups were kept together during the day in the rangeland, while separated accordingly in the evening. On days 15, 10, and 5 prior to exposure to the males, all goats were subject to an ultrasonographic scanning (US) to confirm the anestrus status. In addition, two days after the US-3, all goats received a single intramuscular injection of progesterone (20 mg; Fort Dodge, DF, Mexico) in order to reduce the occurrence of short luteal cycles. Thereafter, goats of the NEO and PEO groups received the same supplementation schedule during a 30 d post adaptation period from 1–30 May. 

### 2.5. Male-to-Female Interaction: The Quest for the Male Effect 

After the 10 d adaptation period, goats continued the supplementation schedule during another 30 d period. Goats from the three treatments were exposed to a 10 d experimental breeding period (20–30 May) with six sexually experienced mix-breed dairy adult bucks (Alpine-Saanen, two per treatment, 3 to 4 years old) of proven fertility and libido. Males were kept in a ruffed cement floor pen (6 × 6 m) before breeding, with free access to alfalfa hay, water, and a mineral mix. Previous to the contact with females, all bucks received an intramuscular injection of testosterone (50 mg, testosterone, Lab Brovel, DF, Mexico) every 3 d × 3 weeks before the male-to-female interaction [20]. Thereafter, bucks were kept in contact with the experimental female groups every day from 1900 to 0800 h (night-time breeding).

### 2.6. Ultrasonographic Evaluation of the Ovary Function and Structures 

The experimental breeding period started late in May and lasted 10 days. Daily incidence of goats showing either estrus signs or copulation was recorded and defined such behaviors as the manifestation of ovulation. Estrus signs were quantified for one hour twice per day (0800 and 1900 h) during the 10 d experimental breeding period. The interval between the onset of joining and occurrence of estrus was also recorded. As mentioned, a total of three ultrasound scanning were performed to confirm the anestrus status of goats prior to male exposure throughout a transrectal real-time B mode USS (Aloka SSD 500 Echo Camera, Overseas Monitor Corp. Ltd., Tokyo, Japan). Then, males were removed from the experimental breeding after 10 days of male-to-female interaction; at this time, the *Opuntia* supplementation schedule concluded. Then, on day 10 post-male removal (10 June), a fourth US was performed to quantify the ovulation rate, measured as the number of corpora lutea present in each ovary. All the US were performed by the same skilled operator. Ovaries were visualized at an image magnification of 1.5×, and the number and diameters of both follicles and corpus luteum observed in each structure were recorded and measured, as previously outlined [25]. The corpus luteum was identified on gray scale as a hypoechoic area within each ovary; ultrasonographic images were also recorded for retrospective analyses. Ovarian function considered the response variables: Estrus induction percentage (EST, %), estrus latency (ESL, hours), and ovulation rate (OR, units). 

### 2.7. Intermittent Blood Sampling, Serum Progesterone and Blood Analytes Quantification

**Figure 1:**
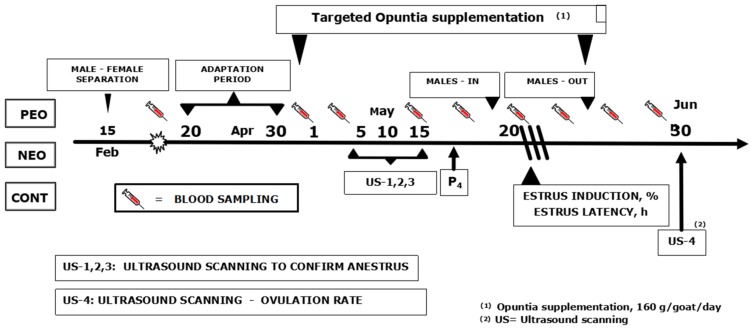
A schematic representation of the experimental protocol of targeted supplementation with *Opuntia megacantha Salm-Dyck* cladodes either protein-enriched (PEO) or non-protein enriched (NEO) as well as not supplemented control (CC) to adult mix-breed (*n* = 45; Alpine-Saanen-Nubian × Criollo) female goats exposed to testosterone treated bucks and to an increased natural photoperiod (Apr–Jun; anestrous season) under semiarid-subtropical rangeland conditions in Northern Mexico (25° N). Note: More details were previously described in the main body of the text.

From 20 April up to 10 June, a twice per week blood sampling was performed to quantify the blood analytes: Total protein (TP), urea (UR), cholesterol (COL), and glucose (GLU). Besides, samples collected from 30 April up to 15 May were quantified by their content of P4 to evaluate the reproductive status of goats. Blood samples (10 mL) were collected by jugular venipuncture from all goats to evaluate blood metabolite concentrations; the analytes were all measured throughout spectrophotometric analyses (Coleman 15 Junior II, Coleman Instruments Division, Perkin Elmer Corp, Sugar Land, TX, USA). Serum TP concentrations were determined in duplicate by using a commercial kit based on the bicinchononic acid reagent considering to bovine serum albumin 16 as standard and performed, as described in the manual kit (Pierce Chemical, Rockford, IL, USA). Serum GLU analyses were also conducted in duplicate throughout spectrophotometer techniques, following protocols supplied by the kit manufacturer (Roche Diagnostic Systems, Inc., Pleasanton, CA, USA). In addition, both serum UR and COL analytes were also measured in duplicate; serum UR concentration was quantified throughout the 640-A kit, based on the urease-18 (Sigma-Aldrich Co., St. Louis, MO, USA), while serum COL concentrations were analyzed throughout the EnzyChrom^TM^ kit (ECCH-100, Bioassay Systems, Hayward, CA, USA); assays were carried out following the protocols outlined by the manufacturer. Serum progesterone concentrations were determined by RIA using a commercial kit (Coat-A-Count, Diagnostic Products Corp., Los Angeles, CA, USA), modified and validated for use in goat serum [6]. The intra and inter-assay coefficients of variation were 6% and 7%, respectively. The anestrus status previously diagnosed with the ultrasound analyses in all goats from the three experimental groups was confirmed from the goats depicted serum P4 concentrations less than 0.5 ng mL^−1^. A schematic representation with the main activities performed during the experimental protocol is shown in Figure 1.

### 2.8. Statistical Analyses 

The response variables LW, BCS, serum TP, UR, GLU, and COL concentrations throughout the experimental period, were determined by split-plot ANOVA for repeated measures across time. Previously, all blood analytes were log transformed because they were not normally distributed. The models included treatment in the main plot, which was tested using animal within treatment as the error term. Time and the time × treatment interaction were included in the subplot and were tested by using the residual mean square, PROC MIXED [26]. In the case of a significant treatment effect, mean separations were achieved using the PDIFF option of the PROC-GLM. Data regarding estrus induction and ovulation rate were analyzed by categorical procedures using the GENMOD procedure of SAS with the LOGIT function. The only effect included in the model was the supplementation treatment, with each animal as a single experimental unit. When significant differences were found among treatments, the LSMEAN/DIFF procedure of SAS (version, company, city, state abbrev if USA, country) was used to compare the mean values. Analysis of variance (PROC GLM; SAS) was performed to evaluate estrus latency; the protected LSD procedure was used to compare means. All the analyses were computed through the procedures of SAS (SAS Inst. Inc. Version 9.4, 2016, Cary, NC, USA); the significance level was set at *p* < 0.05.

## 3. Results

At the beginning of the experimental breeding, no differences among treatments were observed for either LW (*p* > 0.05) or for BCS (*p* > 0.05). In addition, none of the blood analytes differed (*p* > 0.05) among treatments considering neither regarding the overall averages; GLU; 98.44 ± 8.6 mg dL^−1^, UR; 47.71 ± 2.54 mg dL^−1^, COL; 159.77 ± 8.22 mg dL^−1^, and TP; 6.02 ± 0.41 g dL^−1^, nor concerning the treatment × time interaction. Similarly, neither LW nor BCS at the end of the experimental period differed among treatments. Yet, and quite interesting, an increased (*p* < 0.05) estrus induction percentage (100, 57, 42 ± 0.16%), an augmented estrus latency (62, 60, 32 ± 4.2 h), and an enlarged ovulation rate (1.33, 0.71, 0.43 ± 0.23 units) occurred in the PEO and NEO vs. CONT experimental groups, respectively. A summary of data regarding the response variables according to the experimental treatments is presented in Table 2. 

## 4. Discussion 

According to our working hypothesis, the three central queries we pursued to answer in this study were: (1) Does the *Opuntia* targeted supplementation induce the estrus response of anestrous goats exposed to the male effect during the natural non-breeding season? (2) Does the *Opuntia* targeted supplementation promote a differential serum concentration of selected blood analytes in such anestrous goats? (3) Does the *Opuntia* targeted supplementation promote a differential ovarian response regarding estrus latency and ovulation rate of those previously anestrous once exposed to the male effect? With respect to the first question, our results prove that the PEO experimental group depicted 100% estrus induction; the NEO and CONT groups showed 57% and 43% EI, respectively. Interestingly, such increased EI denoted by the PEO group did not parallel differences among treatments regarding serum concentrations of glucose, cholesterol, total protein, and urea, giving essential information to solve the second request. Concerning the third question, all the males linked to each experimental group were able to induce reactivation of ovarian function throughout the stimulus of the “male effect”, yet it was the longest estrus latency, while the largest ovulation rate favored to the PEO followed by the NEO and the CONT experimental groups. 

Therefore, this study unveils a positive stimulus of the *Opuntia effect*, although non-dependent upon increases in either live weight nor in body condition, promoting a significant ovarian upshot not only with respect to estrus induction, but also regarding estrus latency and ovulation rate of these previously anestrous goats strategically supplemented with *Opuntia* cladodes and exposed to the male effect. Moreover, such a physiological scenario was observed in goats managed under grazing conditions during the dry season, facing an increased photoperiod (April to June; 25° N) and facing quite high environmental temperatures (>42°). These observed results are in line with previous findings in which the so-called “*Cactus Effect*” or “*Opuntia Effect*” exerted a positive action upon the reproductive outcomes without increases in live weight in sheep [27,28,29]. In fact, precedent reports have defined a positive effect of cladodes supplementation upon both growth and developmental competence of preovulatory follicles as well as the ovulation rate in sheep [5,27,28,29]. However, when compared with the supplementation of *Opuntia* cladodes + soybean, commercial concentrate and soybean meal, the cactus cladodes-group depicted non-different reproductive outcomes in fat-tailed Barbarine ewes [5]. In lactating sows, intake of spineless cactus reduced blood glucose levels, generated a greater daily feed intake, while it lowered the body weight loss at the end of lactation, reducing the weaning-to-estrus interval length [30]. As previously established, nutritional supplementation increases female responsiveness to the male effect [19] so goats supplemented with high energy diets showed an augmented pattern in LH frequency, generating an increased proportion of depicting estrus in response to the male effect [10]; such a physiological scenario may have been exerted by the PEO-females once exposed to the male effect.

Besides, according to our results, the male effect was able to successfully invoke neurophysiological pathways able to reactivate out-of-season ovarian follicular and luteal cascades, inducing and enhancing the ovarian function while observing the maximal expression of the male effect in those goats supplemented with protein-enriched *Opuntia* cladodes. As previously reported, the male effect upholds the consensus of diverse hints, including olfactory, auditory, visual, and tactile behavioral cues that work in a synergistic fashion to induce the onset of a new reproductive cycle in previously anestrous females; this out-of-season estrus induction process has been termed as an “annual recurrent puberty” [31]. The male effect, throughout diverse sensory clues merged with the neurochemical pheromone hint, accelerates the pulsatile GnRH release, activating in turn, the HPG axis with the concomitant development of ovulatory follicles leading to ovulation as the end-result [32,33]. In this respect, the male effect, a pheromone-chemo signal mediated process, is the undeniable initiator of the GnRH pulse generator activation of previously anestrous females, with an active involvement of the main olfactory system [34,35]. Such male-pheromone cue is elated at the hypothalamic level through the medial nucleus of the amygdala towards the arcuate nucleus that contains kisspeptin neurons [33,36] inducing the activation of the GnRH pulse generator through a neurokinin B-mediated mechanism [37]. While diverse ethyl-branched aldehydes and ketones have been recognized as constituents of this pheromone-based mechanism, it has been proposed to the 4-ethyloctanal as a major player in the activation of the GnRH pulse generator [35]. 

Regarding the blood metabolites, a clear relationship has been proposed among energy balance, metabolic fuel availability, and reproductive fitness [10]. For this reason, any change in the circulating concentrations not only in metabolic hormones, but also in blood metabolites are extremely important cues that transfer information to the central nervous system concerning the nutritional status of animals [38,39]. Therefore, if they want to succeed from a reproductive stand-point, animals must have the ability to align such metabolic status to a correspondent degree of reproductive function, activating in this case, the HPG axis [10]. Undoubtedly, not only the endocrine system, but also a myriad of diverse neural circuitry is triggered in response to the metabolic status as well as in reaction to the circulating levels of specific blood metabolites and/or metabolic hormones [10,38]. Nonetheless, despite the positive ovarian outcomes observed in the *Opuntia*-supplemented dose, none of the analyzed blood metabolites differed among treatments, suggesting that other biomolecules, alternative blood analytes, and metabolic hormones could be related to the increased estrus induction and augmented ovarian performance observed in the protein-enriched *Opuntia* cladode supplemented group. When reviewing the observed average concentration of the quantified blood analytes in this study, they are in close agreement to the reference values reported in goats: Glucose, 2.78–4.16 mmol L^−1^ (50–70 mg dL^−1^), Cholesterol, 2.08–3.38 mmol L^−1^ (80–130 mg dL^−1^), urea 2.09–3.65 mmol L^−1^ (12.6–22 mg dL^−1^), as well as total protein, 61.1–70.1 g L^−1^ [40,41].

In a previous study of our group, neither LW nor BCS or the reproductive variables estrus percentage (89.6%), estrus latency (53.6 h), ovulation percentage (70.3%), ovulation rate (1.07 units), average largest follicle at ovulation (4.5 mm), and the largest corpus luteum (9.6 mm) differed among the *Opuntia* enriched cladodes, *Opuntia* cladodes and Control experimental groups [42]. It is worth mentioning that the opposite outcomes registered in the previous study and the actual study. Nonetheless, of paramount importance is to highpoint that, despite both studies were performed in the same region, in a similar rangeland-based extensive system and the use of similar mixed-breed dairy goat genotypes, the specific microenvironment observed in each agroecological site were certainly different. Unquestionably, the experimental units of the present study depicted not only a better average live weight, but also an increased average body condition score. Besides, although both studies were performed under rangeland-extensive production systems, the former study was carried out in a commercial herd situated far away from the Irrigation District, whereas the actual study was performed in the lower basin of the Nazas River, a region better suited regarding water resources and edaphic quality, different agricultural by-products, and crop residues. Altogether, these specific microenvironment characteristics may have triggered an enhanced reproductive–productive response by making an optimum usage of the targeted cladode supplementation coupled to the male effect in the actual study. 

The action site of the protein-enriched Opuntia cladodes supplementation throughout the HPG axis cannot be established without further research. Although, we still have a fragmentary knowledge regarding the influence of nutritional supplementation as related to follicular growth and ovulation rate, in the ovary, the effect of nutrition unequivocally stimulates follicular growth with concomitant alterations in the insulin-glucose and insulin like growth factor 1-leptin systems, at both systemic and intra-ovarian levels [38,43]. Despite the emergence of follicular waves, it is controlled by the serum concentrations of follicle stimulating hormone, the advance of the follicular growth is only granted in those follicles depicting the lowest sensitivity threshold to FSH since they are able to early-develop LH receptors in the granulosa cells. In turn, such granulosa cell increase in LH receptor expression will cause an amplified activation of the steroidogenic pathway, augmenting the estradiol synthesis, coupled to antrum formation, development of ovulatory follicles, and ovulation [38,43]. An improved nutritional status has been linked to an earlier resumption of ovulation positively related to greater plasma concentrations of insulin, glucose, leptin, and IGF-1 [38,43,44]. These former metabolic players have also been involved as possible amplifiers of the FSH-LH effects at the ovarian level, acting throughout adenosine monophosphate-activated kinase and peroxisome proliferator-activated receptor in the ovary [38]. Moreover, the neuropeptide kisspeptin and its KiSS1R receptor not only govern the onset of puberty, but also the activation of both GnRH and glutamatergic neurons, eliciting ovulation in cycling females while inducing ovulation in anestrous-acyclic females [10,33]. Therefore, bearing in mind these findings and amalgamated with our present results, it is appealing to suggest a possible involvement of systemic and intra-ovarian alterations in the insulin-glucose, the IGF-leptin as well as the kisspeptinergic–glutamatergic systems in those goats supplemented with protein-enriched *Opuntia* cladodes, generating in such a way, not only an increased estrus induction, but also an enlarged estrus latency and an augmented ovulation rate. Undeniably, however, this tempting possibility is yet to be experimentally proven.

To conclude, peri-breeding protein enriched *Opuntia* cladodes supplementation of anestrous goats exposed to active males promoted an increased estrus induction, estrus latency, and ovulation rate. No differences among experimental groups were observed regarding live weight, body condition score, and the serum concentrations of glucose, cholesterol, urea and total protein nor concerning the treatment × time interaction. Further, these thought-provoking results highlight the importance of the bio-fortification of *Opuntia* cladodes process as an interesting management tool to enhance the reproductive outcomes of anestrous goats during the natural non-breeding season, supporting, in this way, to the sustainability of marginal goat production systems under dryland conditions.

## Figures and Tables

**Table 1:** Mean chemical composition (SD), dry-basis, of *Opuntia megacantha Salm-Dyck* cladodes either portein-enriched (PEO), non-protein enriched (NO), and offered as supplement to adult mix-breed (Alpine-Saanen-Nubian × Criollo; *n* = 45) female goats exposed to testosterone treated bucks and to an increased natural photoperiod (Apr–June; anestrous season) under semiarid-subtropical rangeland conditions in Northern Mexico (25° N).

Components	NEO, Fresh	NEO, Dry	PEO, Fresh	PEO, Dry
DM, %	12.9	92.1	12.5	92.0
CP, %	6.4	4.9	29.8	20.5
NDF, %	21.3	14.7	18.3	17.5
ADF, %	19.7	11.9	16.6	17.9
NFC, %	43.8	53.3	24.4	33.9
TND, %	53.1	61.0	57.2	56.4
NEm, Mcal/kg DM	1.9	2.3	2.3	2.2
Ash, %	27.9	24.7	25.5	26.7

NEm was calculated using equations proposed by the National Research Council (2007).

**Table 2:** Least square means regarding live weight (kg) body condition score (units), serum blood metabolites, and ovarian traits in adult mix-breed (Alpine-Saanen-Nubian × Criollo; *n* = 45) female goats supplemented with *Opuntia megacantha Salm-Dyck* cladodes either natural (NEO) or protein-enriched (PEO) or non-supplemented control (CON). Adult goats were exposed to testosterone treated bucks and faced an increased natural photoperiod (Apr–June; anestrous season) under semiarid-subtropical rangeland conditions in Northern Mexico (25° N) **^(1)^**.

Variables	NEO	PEO	CON	S.E. ^(2)^
LW-initial, kg	43.9 ^a^	44.5 ^a^	45.1 ^a^	1.6
BCS-initial, units	2.6 ^a^	2.6 ^a^	2.6 ^a^	0.10
LW-d30, kg	43.9 ^a^	43.8 ^a^	45.4 ^a^	1.7
BCS-final, units	2.5 ^a^	2.5 ^a^	2.6 ^a^	0.20
Glucose, mg/dL	102.0 ^a^	98.1 ^a^	96.6 ^a^	8.7
Cholesterol, mg/dL	158.1 ^a^	165.1 ^a^	156.1 ^a^	8.2
Total protein, g/dL	6.2 ^a^	5.9 ^a^	5.9 ^a^	0.4
Urea, mg/dL	49.1 ^a^	46.5 ^a^	46.9 ^a^	2.4
Estrus induction, %	57 ^b^	100 ^a^	43^b^	12
Estrus latency, h	60 ^a^	62 ^a^	32 ^b^	4.1
Ovulation rate, units	0.71 ^b^	1.33 ^a^	0.43 ^b^	0.20

^(1)^ While no differences among treatments occurred regarding serum blood metabolites (*p* > 0.05), ovarian traits differed among experimental groups (*p* < 0.05). ^(2)^ Most conservative standard error is presented. Different superscripts within variable, show differences (*p* < 0.05).
